# The degenerate coding of psychometric profiles through functional connectivity archetypes

**DOI:** 10.3389/fnhum.2024.1455776

**Published:** 2024-09-10

**Authors:** Simone Di Plinio, Georg Northoff, Sjoerd Ebisch

**Affiliations:** ^1^Department of Neuroscience Imaging and Clinical Sciences, “G. d’Annunzio” University of Chieti-Pescara, Chieti, Italy; ^2^Mind, Brain Imaging and Neuroethics Research Unit, Institute of Mental Health Research, Royal Ottawa Mental Health Centre, Ottawa, ON, Canada; ^3^Institute for Advanced Biomedical Technologies (ITAB), “G. d’Annunzio” University of Chieti-Pescara, Chieti, Italy

**Keywords:** brain-behavior degeneracy, resting-state functional connectivity, self-organizing maps (SOMs), neural redundancy, functional connectivity archetypes, cognitive and behavioral traits, brain-behavior relationships

## Abstract

**Introduction:**

Degeneracy in the brain-behavior code refers to the brain’s ability to utilize different neural configurations to support similar functions, reflecting its adaptability and robustness. This study aims to explore degeneracy by investigating the non-linear associations between psychometric profiles and resting-state functional connectivity (RSFC).

**Methods:**

The study analyzed RSFC data from 500 subjects to uncover the underlying neural configurations associated with various psychometric outcomes. Self-organized maps (SOM), a type of unsupervised machine learning algorithm, were employed to cluster the RSFC data. And identify distinct archetypal connectivity profiles characterized by unique within- and between-network connectivity patterns.

**Results:**

The clustering analysis using SOM revealed several distinct archetypal connectivity profiles within the RSFC data. Each archetype exhibited unique connectivity patterns that correlated with various cognitive, physical, and socioemotional outcomes. Notably, the interaction between different SOM dimensions was significantly associated with specific psychometric profiles.

**Discussion:**

This study underscores the complexity of brain-behavior interactions and the brain’s capacity for degeneracy, where different neural configurations can lead to similar behavioral outcomes. These findings highlight the existence of multiple brain architectures capable of producing similar behavioral outcomes, illustrating the concept of neural degeneracy, and advance our understanding of neural degeneracy and its implications for cognitive and emotional health.

## 1 Introduction

The investigation of optimal configurations of the brain’s functional profiles for specific cognitive traits or behaviors has garnered significant attention in neuroscientific research. Functional connectivity, which refers to the temporal correlation between spatially remote neurophysiological events, plays a crucial role in understanding how different brain regions share information to support cognition (Wu et al., 2021). Researchers have leveraged resting-state functional connectivity (RSFC), measured through techniques like functional magnetic resonance imaging (fMRI), electroencephalography (EEG), or magnetoencephalography (MEG) to predict outcomes such as intelligence, sustained attention, and creative ability (Reineberg et al., 2015; Yoo et al., 2018; Wei et al., 2014; Takeuchi et al., 2012). By analyzing the patterns of functional connectivity, neuroimaging studies aim to uncover relationships between neural network configurations and individual differences in behavioral and cognitive functioning (Geerligs et al., 2015; Tompson et al., 2018). In other words, these studies seek to unveil the *brain-behavior code*, which refers to the complex relationships and interactions between neural connectivity patterns and behavioral outcomes. RSFC can be effectively used to infer psychometric measurements of cognitive abilities, emotions, and personality traits, such as anxiety in young adults (Cermakova et al., 2021) and the sense of agency in psychopathological predispositions (Di Plinio et al., 2019; Berberian et al., 2012), and to distinguish between healthy and diseased populations (Geerligs et al., 2015; Amico et al., 2017; Thijssen and Kiehl, 2017). Thus, by studying the brain-behavior code, researchers can uncover the neural mechanisms that underlie individual differences in behavioral outcomes, dysfunctional psychophysiological states, and cognitive changes throughout the lifespan (Geerligs et al., 2017).

RSFC can help studying the role of intrinsic functional architecture in relation to various personality traits, such as the Big Five traits quantified by the NEO Personality Inventory-Revised (Adelstein et al., 2011). For example, Hsu and colleagues showed that RSFC can predict neuroticism and extraversion in individuals, especially in primary motor and sensory regions (Hsu et al., 2018). A comprehensive study utilized multiple seed regions in major resting-state networks and found widespread associations between RSFC and Big Five personality traits (Markett et al., 2018). RSFC has also been a topic of interest in cognitive neuroscience, for instance showing that specific brain regions, such as the posterior cingulate cortex (PCC) and ventral anterior cingulate cortex (vACC), exhibit distinct activity patterns during resting states compared to cognitive tasks (Greicius et al., 2002). More recently, RSFC has been used for individual trait prediction, indicating its potential in understanding individual cognitive characteristics (Sui, 2022). RSFC studies also significantly contributed to the understanding of personality disorders. For example, these studies reveal that borderline personality disorders have been associated with disrupted connectivity within key brain networks. Specifically, research indicates decreased connectivity in the default mode network (DMN) and salience network (SN), which correlates with the clinical features of borderline personality disorder (BDP) such as emotion dysregulation and impulsivity (Quattrini et al., 2019; Wolf et al., 2011). Further, abnormal connectivity patterns in the fronto-limbic regions, particularly involving the amygdala and anterior cingulate cortex, have been linked to the emotional and cognitive disturbances observed in BDP patients (Krause-Utz et al., 2014).

Even if RSFC stands out as a remarkable predictor of behavior, the variability in such predictions is high, posing relevant trustability challenges for researchers. For instance, a comprehensive study found no consistent predictability for most of the Big Five personality traits except for Openness, suggesting limitations in using RSFC for reliable personality trait prediction (Dubois et al., 2018). Time-frequency analyses revealed significant temporal variability in connectivity, complicating the assessment of stable traits (Chang and Glover, 2010). The neural coding of cognitive functions, particularly fluid and crystallized intelligence, also presents varying evidence regarding specific brain regions, with an unclear contribution of prefrontal, anterior cingulate, and subcortical structures (Jiang et al., 2023; Xue and Xu, 2019; Zamroziewicz et al., 2016). The conflicting or partial evidence in the literature regarding brain-behavioral coding might be explained by the existence of degeneracy in the brain-behavioral code. Degeneracy, in the context of brain encoding of behavior, is the natural consequence of neuroevolutionary trajectories (Di Plinio and Ebisch, 2020; Gould, 2002; Dunbar and Shultz, 2007) and allows the emergence of multiple fundamental neural patterns or structures that could underpin diverse behavioral and cognitive phenotypes. We use the terms *“archetypal patterns”* or” *archetypal connectivity profiles”* to refer to distinct patterns of brain connectivity that represent the most typical or characteristic configurations observed in the sample. These profiles can be identified through machine learning techniques and serve as reference points for comparing individual variations in brain connectivity. In our view, archetypes represent multiple, equally valid neural configurations that can support similar or diverging cognitive functions or personality traits.

Although it is crucial in understanding the brain’s ability to produce diversified behavior through multiple neural pathways or mechanisms (Di Plinio and Ebisch, 2020; Wit and Matheson, 2022), degeneracy is seldom investigated due to its complexity. Nevertheless, degeneracy plays a significant role in neural processes, for example allowing different circuits within the brain to contribute to the same task (Kamaleddin, 2021). Studies showed that degeneracy in brain network functionality contributes to the brain’s functioning in the face of perturbations and enables to compensate to redistribute functions across different regions or circuits, highlighting the brain’s resilience and adaptability (Fornito et al., 2015; Vergotte et al., 2018; Dodel et al., 2020). Neural degeneracy has also been associated with neural plasticity, which is essential for many cognitive functions (Rotondo and Bieszczad, 2019), and with the emergence of baseline neural properties and plasticity profiles. This evidence emphasizes the importance to consider heterogeneities in neural properties when studying brain-behavior relationships (Shridhar et al., 2022). Thus, or degeneracy is a form of within-species redundancy in brain-behavior encoding which enables the brain to adapt to changes, recover from damage, and maintain functionality despite variations in its structure or activity.

To address brain-behavioral encoding, advanced methods that can account for degeneracy are essential. One approach involves leveraging Pareto optimality theory to explore trade-offs that lead to the evolution of phenotypes distributed in a portion of the trait-space resembling a polytope, with vertices representing specialists at one of the traits, or archetypes (Cona et al., 2018). By focusing on models aligning with archetypal configurations, researchers can identify patterns of behavior, or neural archetypes, representative of certain traits (Mittas and Angelis, 2020; Whitacre, 2010), which are possibly underpinned by specific evolutionary neurobehavioral trajectories (Di Plinio and Ebisch, 2020). This approach allows for a nuanced exploration of behavioral traits and their relationship to archetypal configurations, providing a more holistic understanding of the complex interplay between neural processes and behavior.

Self-organizing maps (SOMs) have emerged as powerful tools in the analysis of functional connectivity and its relationship to cognitive and personality traits. These unsupervised learning algorithms facilitate the analysis and visualization of high-dimensional data by projecting it onto a lower-dimensional space while preserving the topological relationships within clusters. This capability makes SOMs particularly suitable for exploring complex brain-behavior interactions where traditional model-based approaches might fall short. For instance, SOMs have been used in fMRI studies, to identify activation sites (Ngan and Hu, 1999), and have been shown to outperform traditional methods such as independent component analysis and voxel-wise univariate linear regression (Katwal et al., 2013). Recent studies have further underscored the applicability of SOMs in understanding brain network topologies associated with various psychological conditions and cognitive abilities. For example, these algorithms have been employed to investigate neurofunctional correlates of BDP and revealed altered global network topology in BPD patients (Xu et al., 2016). Similarly, a study used SOMs to identify distinct amygdalar subregions based on their connectivity patterns, highlighting the method’s strength in uncovering functional heterogeneity (Mishra et al., 2014).

Despite these advancements, there remains a need for novel approaches able to explore the degeneracy of brain-behavioral coding as well as the existence of functional archetypes in the brain. This paper aims to build on these foundations by introducing a novel SOM-based method designed to further unravel the complexity of brain-behavior interactions. By incorporating concepts such as degeneracy and archetypal configurations, the proposed method offers an original exploration of behavioral traits and their relationship to neural processes, providing a more holistic understanding of brain-behavior relationships. We analyze data from the HCP dataset (https://balsa.wustl.edu/; Van Essen et al., 2012) using parametrized, unsupervised, finetuned self-organizing maps to achieve a superior understanding of the neurodiversity underlying varying neural architectures that achieve the same behavioral outcomes. The presence of these archetypal frameworks and diverse evolutionary paths could account for the apparent inconsistencies or gaps in the brain-behavioral coding literature, highlighting the complexity and adaptability of neural mechanisms in shaping human behavior and cognition.

## 2 Methods

### 2.1 Data

We used resting-state fMRI and behavioral data from 500 participants (250 females) of the Human Connectome Project (HCP) database (S1200 data release). The HCP data includes cognitive testing and neuroimaging sessions. The cognitive data is collected using standardized batteries such as the NIH Toolbox Cognition Battery and the Penn Computerized Neurocognitive Battery. Neuroimaging sessions capture high-resolution structural and functional connectivity data. The imaging sessions require participants to lie still inside an MRI scanner for extended periods, during which they might be asked to engage in specific tasks or rest quietly. All participants provided their written informed consent, in accordance with the research protocol approved by the Institutional Review Board of Washington University in St. Louis (Marcus et al., 2011; Smith et al., 2013). For detailed information on the HCP protocol, please refer to Van Essen et al. (2013) and Glasser et al. (2013).

### 2.2 MRI data acquisition and preprocessing

MRI scans were collected at Washington University in St. Louis using a customized 32-channel Siemens 3 T scanner. For each participant, four functional images and one anatomical image were acquired. Image acquisition techniques are delineated in Uğurbil et al. (2013). For the resting-state fMRI images, a gradient-echo echo planar imaging (EPI) sequence was utilized with specific parameters: TR = 720 ms, TE = 33.1 ms, flip angle = 52°, FOV = 208 × 180 mm, matrix = 104 × 90, voxel resolution 2 × 2 × 2 mm, 72 slices covering the entire brain, and a multiband factor of 8. Each subject underwent four resting-state scans, each lasting 15 min (1,200 volumes per run).

We used resting-state runs of the HCP dataset which were pre-processed using the ICA + FIX pipeline. The fMRI datasets underwent preprocessing using the HCP’s main pipeline as described by Glasser et al. (2013). This procedure corrects for spatial distortions resulting from gradient nonlinearities through gradient distortion correction. This is followed by motion correction, during which volumes are aligned. Subsequently, the fMRI data is registered to the individual’s structural images using nonlinear registration techniques. A global intensity normalization is then performed to scale the 4D fMRI volume, followed by temporal high-pass filtering to mitigate low-frequency drifts and by the IC + FIX pipeline created by the HCP project (Glasser et al., 2013). The ICA + FIX pipeline is a significant component of the Human Connectome Project data processing strategy, primarily focused on the analysis of functional magnetic resonance imaging (fMRI) data. ICA stands for Independent Component Analysis, a computational method used to separate a multivariate signal into additive, independent components. FIX, or FMRIB’s ICA-based X-noiseifier, is a tool developed by the Functional MRI of the Brain (FMRIB) Centre at the University of Oxford. FIX is used for the automatic removal of motion and other non-neural artifacts from fMRI data.

With respect to fMRI data, functional connectivity data encompassed the four resting-state scans for each of the subject. The data collected from these sessions was processed and analyzed to construct brain functional connectomes. First, the connectivity matrices for the cortical regions were extracted from the FC dataset. Connectomes of 346 × 346 nodes for each single subject, and for each resting-state run, were constructed using cortical atlas by Joliot et al. (2015). The extracted connectivity matrices were concatenated across all resting-state scans, resulting in a comprehensive dataset of brain connectivity measures. Following common practices, and to reduce data skewness, we determined functional connectivity using the z Fisher transform of the Pearson correlation among preprocessed time series.

To reduce the dimensionality of the high-dimensional connectivity data, and avoid redundant information, Principal Component Analysis (PCA) was performed before clustering. This technique has already been used successfully in neuroimaging studies (Margulies et al., 2016; Di Plinio and Ebisch, 2018). The lower triangular part of each connectivity matrix, excluding the diagonal, was extracted and flattened into a vector. These vectors, that are unique connectivity values, served as the input for the PCA. A parallel analysis (Horn, 1965; Ledesma and Valero-Mora, 2007) was implemented through 10,000 simulated eigenvalues using a Monte Carlo procedure which started from random datasets with equal structure as the original dataset. Then, the number of factors to retain in the factor analysis was selected as the number of eigenvalues above the 95th percentile of the null distribution. The parallel analysis determined that 14 principal components should be retained. The scores of these principal components were standardized (z-scored) to produce the reduced dataset, which was subsequently used for clustering with Self-Organizing Maps (SOM).

### 2.3 Behavioral data preprocessing

The HCP database provides a comprehensive suite of psychometric measures that are essential for delineating the core functions necessary to understand the relationship between brain and behavior. These psychometric tools are meticulously designed to assess a wide array of cognitive, socioemotional, and behavioral functions, thereby offering a robust framework for investigating the underlying neural processes of interest. Specifically, our study utilized measures including personality traits as quantified by the Big Five questionnaire (covering agreeableness, extraversion, neuroticism, openness, and conscientiousness); discounting behavior, which assesses the propensity to devalue delayed rewards; and socioemotional measures, such as self-reported fear, anger, sadness, positive affect, life satisfaction, sense of meaning and purpose, friendship, loneliness, perceived hostility, perceived stress, and self-efficacy. The following cognitive scores were also incorporated: overall cognitive performance, fluid intelligence, and crystallized intelligence. Finally, motor and physical scores included were: endurance, gait speed, dexterity, and strength. These psychometric measures were rigorously preprocessed using Box-Cox correction to improve normality and subsequently normalized to a range of 1 to 100. This normalization procedure was adopted for comparability across different psychometric dimensions and enhancing the interpretability of the results. By aligning the normalized behavioral scores with the dimensionality of the brain connectivity data across multiple resting-state scans, we ensured a robust and integrative approach to analyzing the intricate relationships between neural connectivity patterns and diverse psychometric outcomes.

### 2.4 Parametrization of self-organizing maps (SOM)

Self-Organizing Maps (SOMs; Kohonen, 1990, 2013) are a type of artificial neural network used for unsupervised learning. SOMs are particularly effective for clustering and visualizing high-dimensional data by mapping it onto a lower-dimensional grid, typically two-dimensional. This mapping process preserves the topological properties of the input data, ensuring that similar data points are placed close to each other on the grid. Each neuron in the SOM represents a weight vector with the same dimensions as the input data. For each input data point, the algorithm identifies the neuron with the closest weight vector, known as the Best Matching Unit (BMU). The BMU’s weight vector, as well as those of its neighboring neurons, are then updated to become more similar to the input data point. This process is repeated over many iterations (epochs), gradually refining the map. SOMs are valued for their ability to perform vector quantization and dimensionality reduction. These capabilities make them suitable for various applications, including pattern recognition, data compression, and feature extraction.

To determine the optimal SOM parameters, we conducted a double-step finetuning comprehensive parametrization process, evaluating different SOM dimensions and epochs. This parametrization process ensures a thorough exploration of the SOM parameter space, identifying the optimal settings for achieving stable and accurate clustering of the reduced brain connectivity data. SOM dimensions refer to the layout and size of the grid that forms the SOM. Typically represented in a two-dimensional array, these dimensions define the number of neurons (or clusters) and their arrangement in the map, influencing the granularity and resolution of the clustering. Epochs, in the context of SOM clustering, represent the number of times the training dataset is presented to the network during the training process. More epochs typically lead to better convergence, allowing the SOM to adjust its weights more thoroughly and produce more accurate and stable clustering results, but can also lead to overfitting, so that the process should be accurately supervised.

In the first finetuning step, we explored the stability of many SOM dimensional grids, from a 2 × 2 grid to a 10 × 10 grid, using a standard set of epoch values (epochs = 100). This preliminary analysis indicated a poor stability of high-dimensional grids (above 4 × 4). In the second step, two low-dimensional SOM grids were considered: 2 × 2 (4 neurons, or clusters) and 3 × 3 (9 neurons, or clusters). For each dimension, we evaluated a range of epoch values, from 40 to 260, in increments of 20. This extensive grid search allowed us to explore the impact of both SOM dimensions and the number of training epochs on the clustering performance. For each combination of SOM dimension and epoch values, the clustering process was repeated 200 times to ensure robust results. The SOM was trained on the reduced data using the *selforgmap* function with the specified parameters. After training, the data points were assigned to clusters. To assess clustering consistency, we computed the Adjusted Mutual Information (AMI) and Adjusted Rand Index (ARI) for all pairs of clustering results across the runs. These metrics evaluate the similarity between cluster assignments, with higher values indicating more consistent clustering. The AMI and ARI values were averaged for each combination of SOM dimension and epoch values. The mean AMI and ARI values were calculated for each combination of SOM dimension and epoch values. Average AMI and ARI values were visualized against the number of epochs for each SOM dimension. This visualization provided a clear comparison of the clustering performance across the parameter grid, highlighting the optimal configurations. The optimal parameters were identified based on the highest ARI and AMI values. Specifically, we identified the best number of epochs for each SOM dimension that maximized clustering stability without falling into overfitting situations. The final parameters were saved for subsequent analyses, and the results were visualized to illustrate the impact of SOM dimensions and epochs on clustering performance.

### 2.5 Consensus clustering procedure

To enhance the robustness and reliability of the clustering results, a consensus clustering procedure was implemented following the iterative application of Self-Organizing Maps (SOM). This procedure aimed to aggregate the results of multiple SOM runs and derive a consensus clustering that is more stable and reliable than individual runs; this process avoided falling into local minima or suboptimal solutions. The parameters for the SOM (i.e., dimensions and epochs) were selected based on the previous parametrization phase. For each combination of SOM dimensions and epochs identified as optimal, the consensus clustering procedure was applied.

For each chosen SOM configuration, the consensus clustering was executed 100 times using the optimal dimensions and epochs chosen after the finetuning procedure described in 2.4. Each run produced a clustering result, and these results were stored. A consensus matrix was constructed to capture the co-assignment frequency of data points across the multiple SOM runs: for each pair of data points, the number of times they were assigned to the same cluster across the 100 runs was counted; this count was then normalized by the total number of runs to obtain the consensus matrix, representing the probability of co-assignment.

To derive the final consensus clustering, the dissimilarity between data points was first calculated as one minus the consensus matrix values. Then, hierarchical clustering using average linkage was applied to the dissimilarity matrix. The final clusters were determined by cutting the dendrogram to form the same number of clusters as the original SOM configuration. To identify the most representative clustering from the multiple SOM runs, we used Adjusted Rand Index (ARI), which was calculated between the consensus clustering and each of the 100 runs. The SOM clustering that achieved the highest ARI and AMI with the consensus clustering was selected as the best run, providing the final clustering. This consensus clustering procedure ensured that the final clustering results were robust and consistent, minimizing the variability inherent in individual SOM runs and providing a more reliable basis for subsequent analyses. The consensus clustering results were finally visualized. Average connectivity matrices for each cluster were computed and visualized, re-ordered based on network assignments for interpretability (Di Plinio and Ebisch, 2018). We also estimated and shown differences between clusters to give a comprehensive picture of the results.

### 2.6 Brain-behavior association analyses

To uncover the degeneracy in the brain-behavior code, a two-dimensional (two-way) ANOVA was employed to analyze the association between brain connectivity clusters (derived from Self-Organizing Maps, SOM) and behavioral factors. This approach allows for the examination of how different SOM clusters (neurons) are associated with behavioral profiles.

SOM clusters (or archetypes) are organized into a two-dimensional grid which represent the SOM dimensions, enabling the representation of each cluster’s location on the grid. These coordinates were used as categorical variables in the subsequent analyses, which used both ANOVA and linear mixed-effects (LME) models in a multiverse analysis framework. The ANOVA and LME models included: main effects: the impact of the X and Y coordinates of the SOM clusters on the behavioral scores; interaction effects: the combined effect of the X and Y coordinates on the behavioral scores. Post-hoc multiple comparisons were performed for each main effect and for the interaction effect. For the interaction effect, comparisons were made between all possible pairs of SOM grid coordinates, but only the comparisons where one factor (either X or Y) differed were considered. We applied both false discovery rate and Bonferroni corrections for multiple comparisons (Benjamini and Yekutieli, 2001; Holland and Copenhaver, 1988) and interpreted data according to modern guidelines using multiple thresholding methods to control both type I and type II errors.

Model statistics and multiple comparison results were computed for each analyzed behavioral factor. This multiverse, comprehensive analysis provided insights into how different brain connectivity patterns, as represented by SOM archetypes, are associated with various behavioral profiles, thus highlighting the potential degeneracy in the brain-behavior code. The methodology outlined above ensures a thorough and robust analysis of the complex relationships between brain connectivity and behavior, leveraging advanced clustering techniques and rigorous statistical testing to uncover meaningful findings.

Data analyses were executed using MatLab version 2022b.^1^

## 3 Results

### 3.1 SOM parametrization

**Figure 1:**
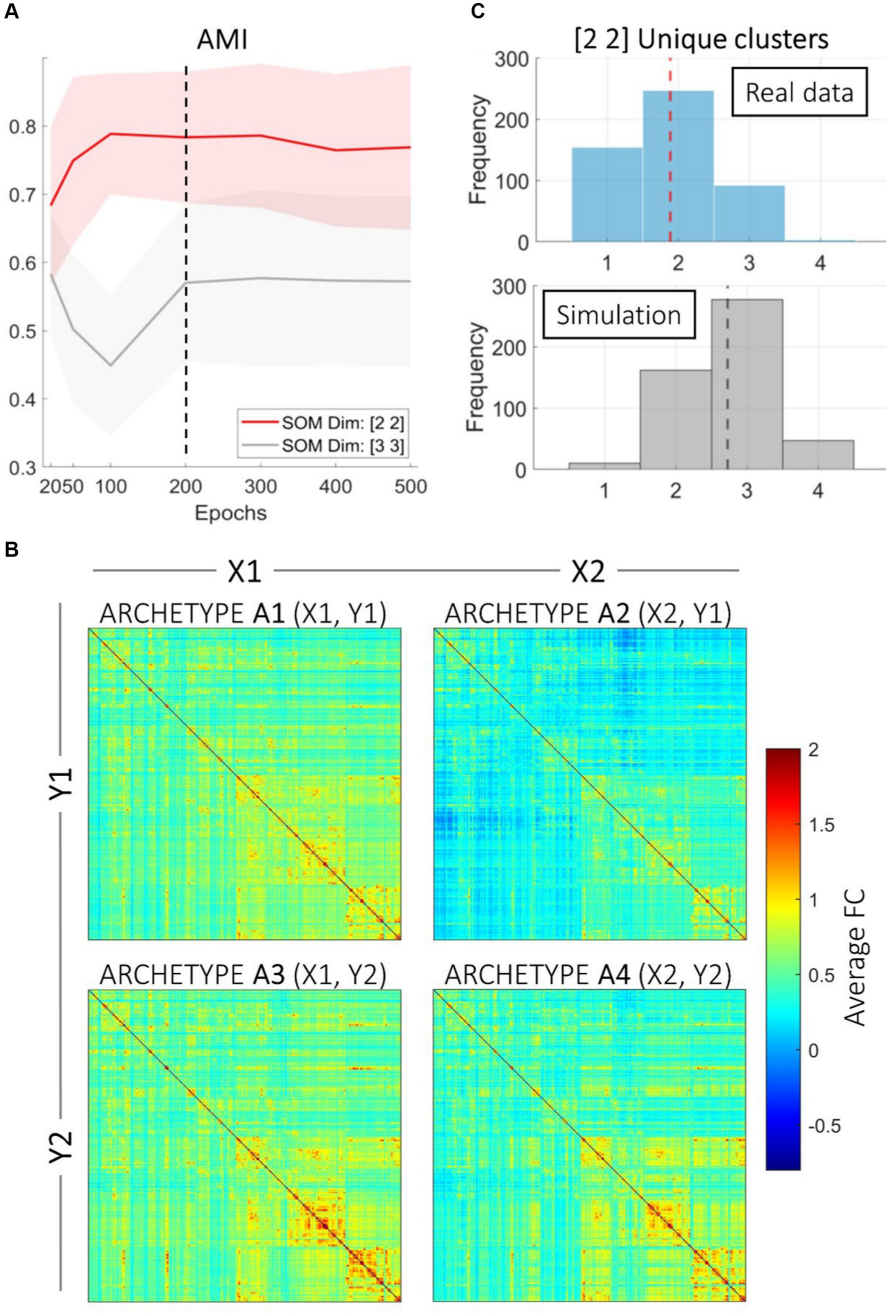
SOM clustering. **(A)** Results of the finetuning process for selecting optimal SOM parameters. Stability of Self-Organizing Map (SOM) clustering, exemplified by adjusted mutual information (AMI) indices is represented across different numbers of epochs for two SOM dimensions: [2 2] and [3 3]. The shaded areas represent the standard error around the mean values. The dashed vertical line at 200 epochs indicates the selected optimal number of epochs. The red line represents the SOM dimension [2 2], while the gray line represents the SOM dimension [3 3]. AMI and ARI values (ARI values not represented) tend to stabilize after 200 epochs, indicating that this is an appropriate choice for the number of epochs to ensure stable clustering results. **(B)** Average connectomes are represented for each archetype derived from the 2 × 2 grid and 200 epochs. The connectomes are represented as heatmaps, where each pixel indicates the average connectivity strength between two brain regions. Color intensity indicates the average strength of Fisher-transformed functional connectivity within each Archetype. **(C)** The top panel shows the distribution of unique SOM clusters assigned per subject. The average number of unique clusters per subject is indicated by the red dashed line. The bottom panel displays the results of the Monte Carlo simulation with random cluster assignment, with the average number of unique clusters per subject indicated by the black dashed line. This comparison highlights the tendency for resting-state functional connectivity runs to be consistently assigned to the same clusters, as opposed to random assignment.

The two-step fine-tuning process for selecting the dimensions of the SOM grid and the number of epochs identified the optimal configurations as small cluster grids (2 × 2 and 3 × 3) with the number of epochs set to 200 (Figure 1A). Both adjusted mutual information (AMI) and adjusted Rand indices (ARI) decreased with an increasing number of epochs. Additionally, lower epochs showed a mismatch between the chosen SOM dimensions. Thus, we employed 2 × 2 and 3 × 3 SOM clustering with 200 epochs on the HCP fMRI resting-state data of functional connectivity. These configurations achieved the best balance between clustering stability and computational efficiency, making it ideal for subsequent analyses.

### 3.2 SOM clustering

We present results using the 2 × 2 dimensional grid as a benchmark not only to ease interpretability, but also to demonstrate the significance of our proposed approach. SOM clustering with the 2 × 2 grid and 200 epochs resulted in 4 (2 × 2) different clusters. Because of the rationale of our study, each SOM cluster connectome is referred to as an *archetype*. We computed the average functional connectivity across subjects for each cluster, displayed in Figure 1B. The four archetypes had different numerosity: Archetype 1 (SOM coordinates = X1, Y1) included 400 participants; Archetype 2 (X2, Y1) included 798 participants; Archetype 3 (X1, Y2) included 314 participants; finally, Archetype 4 (X2, Y2) included 472 participants.

The upper panel in Figure 1C presents a histogram of cluster variability across subjects, highlighting the distribution patterns. Since most subjects are consistently assigned to one or two archetypes, these results demonstrate consistent within-subject clustering. The lower panel in Figure 1C, instead, shows a parallel Monte Carlo analysis where the same number of subjects and runs were simulated with random cluster assignment. This analysis offers insights into how often different resting-state runs of the same subjects are assigned to the same cluster compared to a null (random) assignment. Results indicate that the clustering assignments are different from random, suggesting that connectomes of the same subject tend to be assigned to the same archetype. However, many subjects’ connectomes are assigned to 2 or 3 clusters, indicating substantial interindividual variability in a subject’s connectional profile over time, that is, over subsequent resting-state acquisitions.

**Figure 2:**
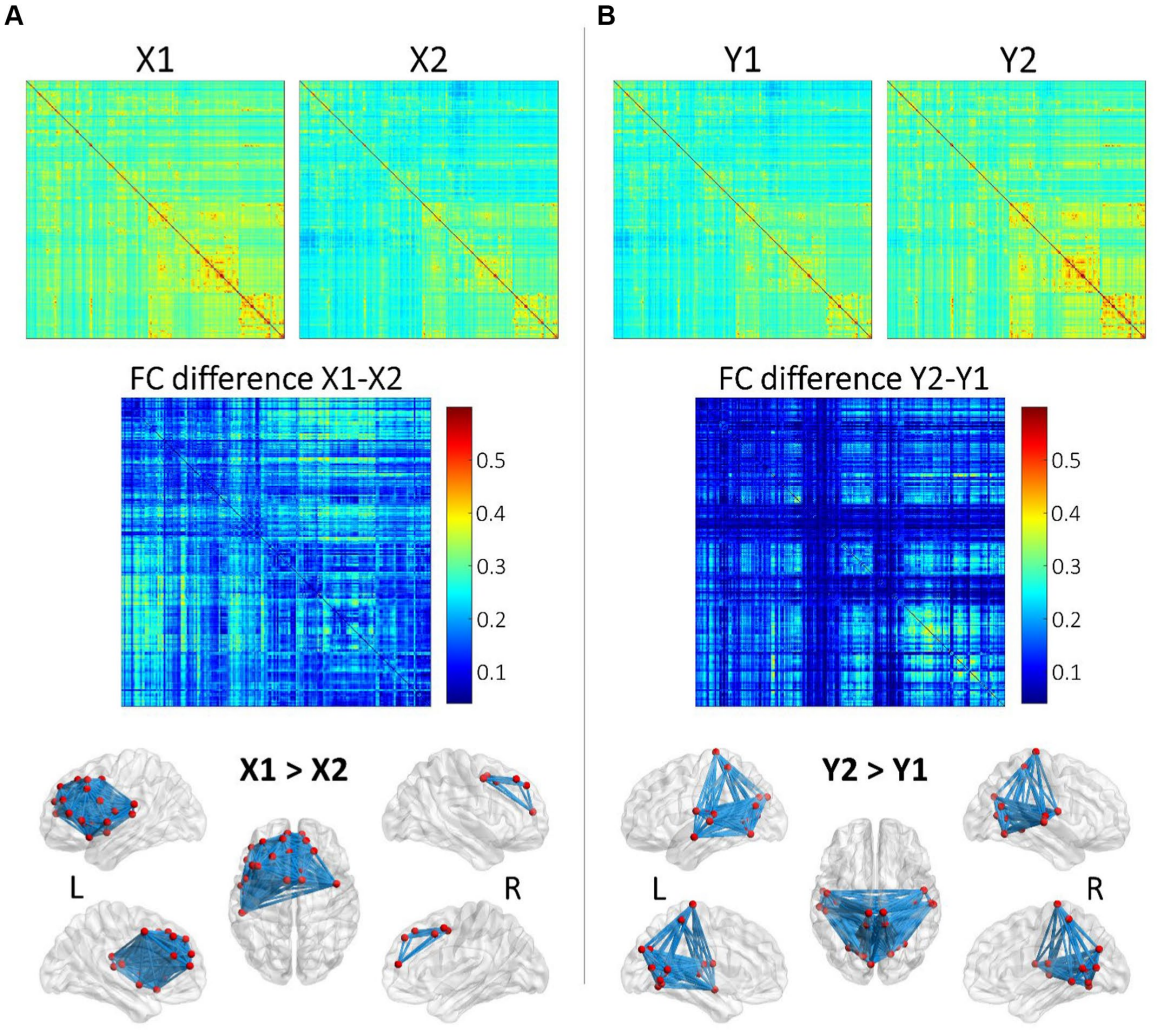
Analysis of average functional connectomes within and between X and Y dimensions. **(A)** Functional connectome (FC) matrices for X1 and X2 and the differences in functional connectivity (FC difference) between these two conditions. The top row displays the FC matrices for X1 and X2, showing the average connectivity patterns across brain regions. The middle row shows the FC difference (X1–X2), where the color intensity represents the magnitude of connectivity differences, with red indicating greater connectivity in X1 compared to X2. The bottom row illustrates these differences in connectivity on a3D brain model obtained using BrainNet Viewer (www.nitrc.org/projects/bnv/; Xia et al., 2013). **(B)** This subfigure follows the same structure as **(A)** but focuses on the Y dimension, comparing Y1 and Y2. The FC matrices for Y1 and Y2 are shown on the top, followed by the FC difference (Y2-Y1) in the middle. The color map indicates regions with greater connectivity in Y2 compared to Y1 (red). The brain models at the bottom illustrate the regions with significantly stronger connectivity in Y2 compared to Y1 for both hemispheres.

**Figure 3:**
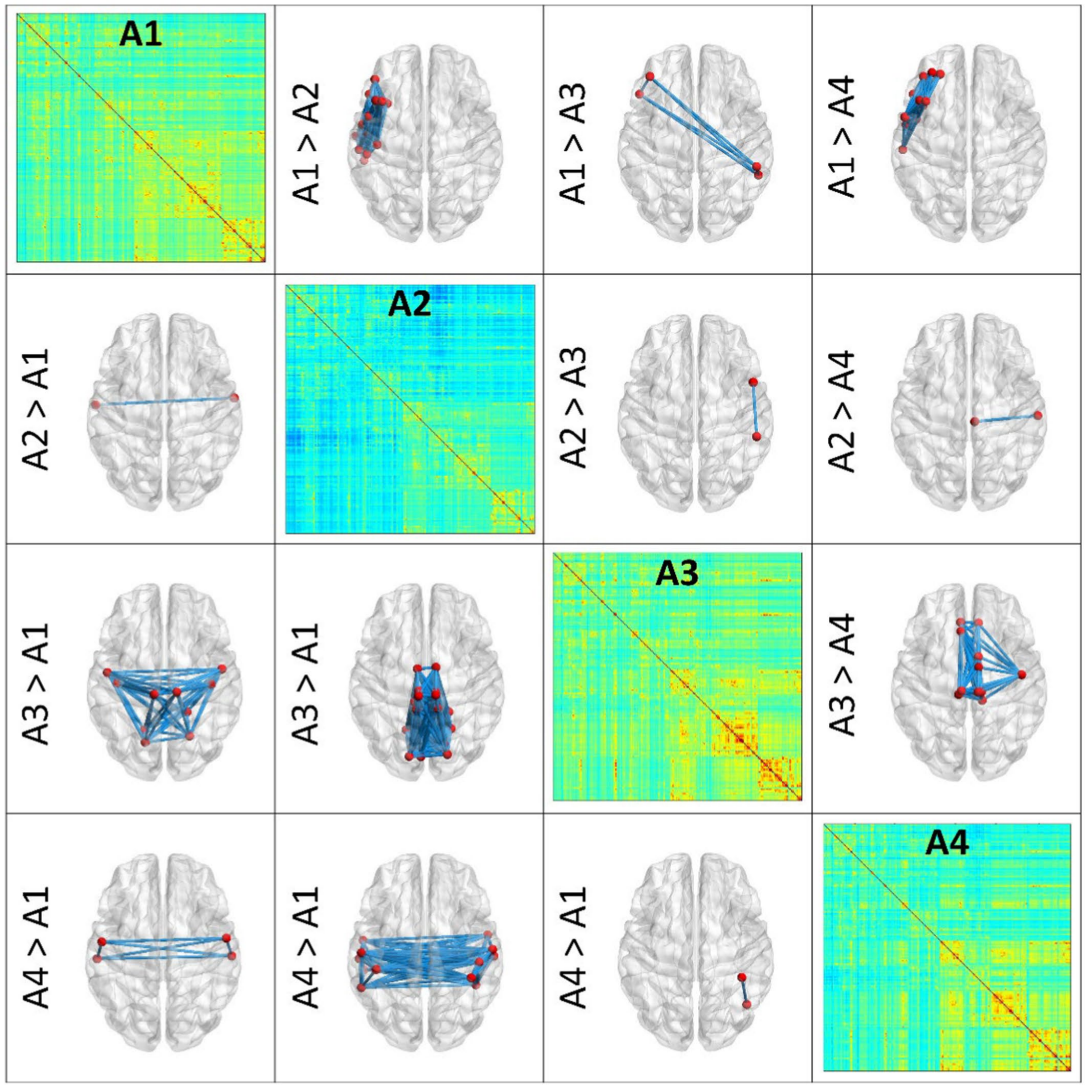
Pairwise differences between single Archetypes (A1, A2, A3, A4). The figure presents pairwise differences in functional connectivity between SOM archetypes through 3D brain renderings, which were obtained using BrainNet Viewer. Archetypes are represented in the diagonal, while differences among couples of archetypes are represented in the lower and upper triangles of the figure. In each brain model, blue lines represent the connections that differ the most between the archetypes. Nodes involved in these connections are depicted in red. These results help characterizing the (average) functional profile of each archetype. Top, anterior; Bottom, posterior.

Pairwise archetypal differences are detailed in Figures 2, 3, providing a comprehensive characterization of each archetype at the connectional level. These findings underscores the subtle differences and unique features of each archetype, enhancing our understanding of the functional connectivity patterns within and between archetypes. More specifically, Figure 2 reports average connectomes within the X and Y dimensions (X1, X2, Y1, Y2), and the differences within each dimension (X1 vs. X2; Y1 vs. Y2). These findings show that the X dimension separates archetypes based on anterior and left-lateralized prefrontal connectivity, while the Y dimension separates archetypes based on posterior, symmetrical connectivity in parietal, occipital, and temporal regions. Similarly, Figure 3 reports differences within specific archetypes. These two figures highlight similarities and differences in functional connectivity patterns within and between X and Y dimensions, providing insights into the neural correlates underlying functional brain archetypes.

### 3.3 Brain-behavioral coding

**Figure 4:**
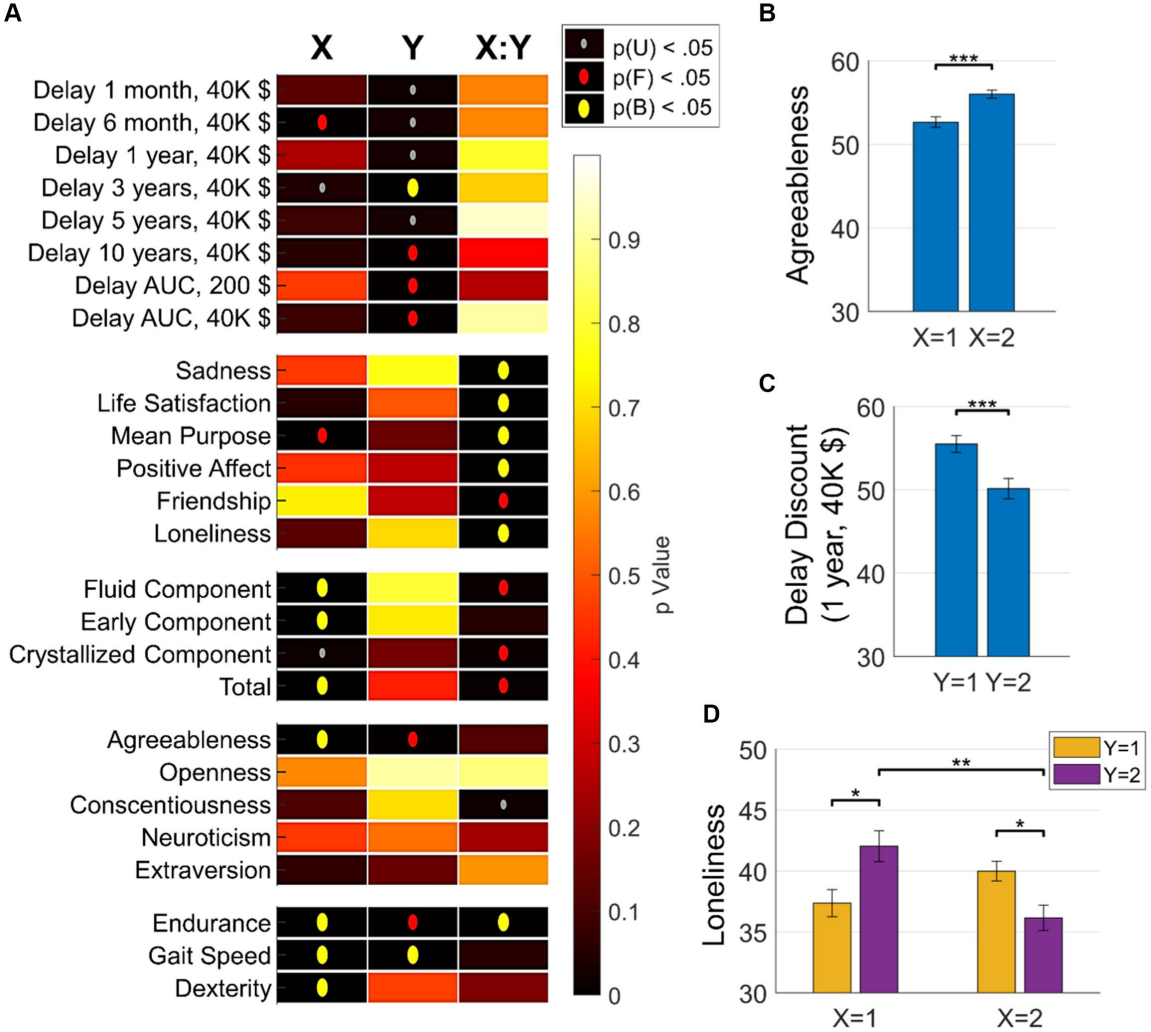
Brain-Behavior associations. **(A)** Associations between brain archetypes (as identified by the 2 × 2 SOM grid) and various behavioral measures. The heatmap shows the significance of these associations across three dimensions: X, Y, and their interaction X:Y. The vertical axis represents behavioral measures, including discounting attitudes, affective measures, socioemotional measures, cognitive abilities, personality traits, and physical abilities. The *p*-value significance is represented by the color intensity, ranging from yellow (high *p* value, less significant) to red (low *p*-value, highly significant). Significant p-values (*p* < 0.001) are indicated by gray dots; *p*-values significant after FDR correction are indicated by red dots; *p*-values significant after Bonferroni correction are indicated by yellow dots. This figure highlights the intricate and selective encoding of various behavioral attributes by different dimensions of functional connectivity archetypes. **(B)** Bar plots show the mean scores and standard errors of various behavioral measures across different clusters identified by the 2 × 2 SOM grid. Each plot compares the effects of the X and Y dimensions of the SOM on specific behavioral attributes, highlighting the complexity and specificity of brain-behavior relationships. Significance markers (after Bonferroni correction): * *p* < 0.05; ** *p* < 0.01; *** *p* < 0.001.

The analysis of functional archetypal coding of psychometric scores yielded several significant insights into the complex way brain functional architectures code for behavioral outcomes (Figure 4A): different SOM dimensions coded for different types of attributes. Specifically, the X dimension of SOM archetypes predominantly coded many psychometric indices such as fluid intelligence, dexterity, and agreeableness. This indicates that the X dimension is linked to a broad range of individuals behavioral facets reflected in cognitive, physical, and personality scores. In contrast, the Y dimension of SOM archetypes mainly coded for discounting behavior, which refers to the tendency of individuals to devalue rewards or benefits that are delayed in time. This indicates that brain connectivity pattern changes in the Y dimension are directly linked to planning and attitudes towards future rewards. Moreover, the interaction between the two SOM dimensions significantly encoded behavioral attributes related to socioemotional traits, such as sadness, life satisfaction, and loneliness. These findings underscore a consistent yet selective encoding of behavioral attributes by archetypes of functional connectivity.

To better understand these results and investigate the degeneracy in brain-behavior encoding, we applied Bonferroni-corrected multiple comparison testing on the analyses. Relatedly, Figure 4B presents selected representative comparisons showing distinctive associations with personality traits (e.g., agreeableness), socioemotional measures (e.g., loneliness), and discounting behavior. Overall, these findings underscore the intricate and selective encoding of behavioral attributes by functional connectivity archetypes. The results illustrate that different dimensions of brain connectivity are linked to various aspects of behavior, highlighting the complexity and degeneracy of brain-behavior relationships. For instance, in the case of loneliness, there are distinctive connectivity profiles associated with higher or lower behavioral outcomes. Specifically, archetypes 2 (X2, Y1) and 3 (X1, Y2) are associated with high scores of loneliness, while archetypes 1 (X1, Y1) and 4 (X2, Y2) are associated with low scores of loneliness.

Importantly, archetypes 2 and 3, which both code for high loneliness, exhibit very different functional connectivity profiles, with generally very high or very low functional connectivity values, respectively. We stress here that these results is impossible to capture with ordinary measures and highlights the potential of the current approach to reveal degeneracy in brain-behavior coding, beyond linear associations of regional or network connections with behavior.

## 4 Discussion

The present study explored the concept of degeneracy in the brain-behavioral code. We developed a new approach integrating Self-Organizing Maps (SOMs) to analyze resting-state functional connectivity (RSFC) and its relationship to psychometric assessments of behavior. Our findings provide significant insights into the complex and redundant nature of brain-behavior interactions, showcasing the brain’s adaptability and robustness. One of the central findings of this study is the identification of distinct archetypal connectivity profiles, or archetypes, that correlate with specific psychometric profiles. The SOM clustering revealed primary archetypes, each exhibiting unique patterns of functional connectivity (Geerligs et al., 2015; Tompson et al., 2018; Wu et al., 2021). These archetypes represent different patterns of brain connectivity that are consistent across subjects. The within-and between-network connectivity patterns of these archetypes revealed distinct functional profiles, supporting the validity of the SOM clustering approach (Xu et al., 2016; Mishra et al., 2014) and supporting the validity of the concept of archetype itself. Notably, the results demonstrate that different brain connectivity patterns can support similar behavioral outcomes, underscoring the concept of degeneracy in the brain-behavior code (Di Plinio and Ebisch, 2020; Whitacre, 2010; Mittas and Angelis, 2020). Our pipeline revealed that the different dimensions of the SOM archetypes were differentially associated with psychometric measurements. The first dimension (X) was predominantly correlated with cognitive and physical behaviors, such as fluid intelligence and dexterity, as well as personality traits like agreeableness. In contrast, the second dimension (Y) was primarily linked to discounting behavior, reflecting individuals’ tendencies to devalue delayed rewards. The interaction between these dimensions was significantly associated with socioemotional traits, including sadness, life satisfaction, and loneliness.

The concept of degeneracy in biological systems, where different structures can produce similar functions, is well-established in neuroscience (Wit and Matheson, 2022; Dodel et al., 2020; Fornito et al., 2015). We extend the concept of degeneracy to brain-behavior relationships, showing two crucial points: first, distinct functional architectures of the brain can be associated with similar behavioral outcomes; second, and more importantly, the brain can achieve behavioral robustness through multiple network configurations. Our findings are consistent with previous research that has demonstrated degenerate functional connectivity patterns within neural networks during emotional experiences (Doyle et al., 2022) as well as with findings highlighting the genetic component underlying shared connectivity profiles across different psychiatric conditions (Moreau et al., 2022). The presence of degeneracy suggests that the brain’s functional architecture is highly adaptable, capable of maintaining functionality despite structural or activity variations pathways (Fornito et al., 2015; Kamaleddin, 2021). This adaptability is crucial for understanding how the brain compensates for damage, supports recovery, and sustains cognitive and emotional health across the lifespan (Rotondo and Bieszczad, 2019).

Our study underscores the importance of considering innovative and multidimensional approaches when studying brain-behavior relationships. Traditional linear models may overlook the complexity and variability inherent in these relationships. The use of SOM and consensus clustering provides a powerful tool to capture and analyze this complexity, offering new insights into the functional organization of the brain (Katwal et al., 2013; Ngan and Hu, 1999). The methodological framework employed in this study has several strengths: first, the iterative SOM and consensus clustering approach ensures robust and stable clustering results, reducing the impact of variability and noise; second, by including multiple connectivity patterns for each participant, we simultaneously characterized interindividual (between-subject) and within-subject differences within brain functional archetypes; third, the linkage of functional connectivity patterns to a wide range of behavioral traits demonstrates the applicability of our approach to understanding complex brain-behavior relationships. Moreover, the robustness of our clustering approach is supported by similar methods used to identify functional connectivity subtypes (e.g., in autism Urchs et al., 2022).

Degeneracy plays a significant role in the brain’s ability to recover from stroke and other vascular insults, which is highly relevant to our study of brain-behavior relationships and neural plasticity. Neural networks exhibit a remarkable capacity for reorganization, where multiple pathways can compensate for damaged regions, thereby supporting functional recovery. Fornito et al. (2015) discussed how degeneracy in neural networks facilitates recovery by enabling multiple neural pathways to compensate for damaged regions. Similarly, Crofts et al. (2011) found evidence of compensatory mechanisms through changes in functional connectivity after stroke, highlighting the role of alternative pathways in neural reorganization. Guggisberg et al. (2014) further demonstrated that the brain’s adaptability through degeneracy is crucial for regaining lost functions, with reorganization influenced by the severity and location of the stroke. Additionally, Carter et al. (2012) emphasized that rehabilitative training can enhance the brain’s ability to utilize redundant pathways for functional recovery. Together with such findings, our study aims to elucidate the complex interplay between brain connectivity and behavior through the lens of degeneracy. By incorporating these insights, we emphasize the critical role of neural redundancy and flexibility in supporting diverse cognitive and behavioral phenotypes, thus advancing our understanding of the brain-behavior code.

Despite the promising results, several limitations should be acknowledged. First, the study’s reliance on RSFC data from a single dataset (HCP) may limit the generalizability of the findings (Smith et al., 2013; Glasser et al., 2013). Second, while our sample size is substantial future research should replicate these analyses using diverse datasets and across different populations to validate the robustness of the identified archetypes and their associated behavioral traits (Shridhar et al., 2022). Additionally, while SOMs are powerful tools for clustering and visualization, they also have limitations, such as sensitivity to parameter selection. The extensive parametrization process used in this study aimed to mitigate these issues, but further refinement and comparison with other clustering techniques could enhance the reliability of the results. Finally, the multidimensional nature of the results, while informative, can complicate interpretation.

As a natural consequence of our seminal, mainly methodological contribution, we expect future studies to delve deeper into degenerate regional and network encoding of behavior. Future research directions include the applications of our methods to other large-scale neuroimaging datasets, such as the UK Biobank and the ABCD Study, to validate SOM-based approaches for the study of degeneracy across diverse populations and age groups. Also developing more intuitive visualization and interpretation tools will help understanding these complex relationships. Moreover, longitudinal studies will be crucial to investigate the stability and evolution of the identified functional connectivity archetypes over time to understand how brain-behavior relationships develop across the lifespan. Finally, future studies may explore the integration of additional statistical mixture approaches, such as Bayesian Kernel Machine Regression (BKMR) and Weighted Quantile Sum (WQS) regression (Invernizzi et al., 2022), to further dissect the complex relationships between brain connectivity and behavior.

Concluding, this study presents a novel framework for understanding the degeneracy in the brain-behavior code, demonstrating that distinct functional connectivity patterns can be associated with similar psychometric profiles. We contribute to the literature by providing empirical evidence for the degenerate coding of behaviors in the human brain. These findings challenge traditional models often assuming linear and region-specific associations between brain and psychometric measures. The use of self-organizing maps and consensus clustering provides a powerful approach for uncovering these patterns, underscoring the importance of considering neural degeneracy and adaptability in cognitive neuroscience. These advanced machine learning techniques can support future research in this exciting area, offering the potential to further understand the complex code underneath brain-behavior interactions and contributing to the development of more effective interventions for cognitive and emotional disorders.

## Data Availability

The original contributions presented in the study are included in the article/supplementary material as well as https://github.com/smndpln/DegeneracyBrainArchetypes. Further inquiries can be directed to the corresponding author.

## Footnotes

^1^https://it.mathworks.com/
